# Supplementary material for: The Systems Biology Research Tool: evolvable open-source software
Source: BMC Syst Biol. 2008 Jun 29;2:55. doi: 10.1186/1752-0509-2-55 (PMC2446383; doi:10.1186/1752-0509-2-55)
Supplement: Additional file 1 — SBRT Archive. An archive of the current version of the Systems Biology Research Tool. [file 1752-0509-2-55-S1.zip › sbrt-1.4.0/doc/developers_guide/api/sbrt/kernel/util/MutableProgressMonitor.html]

MutableProgressMonitor


|  |  |  |
| --- | --- | --- |
| **Overview** \| **Package** \| **Class** \| **Use** \| **Tree** \| **Deprecated** \| **Index** \| **Help** | |  |
| **PREV CLASS**   **NEXT CLASS** | **FRAMES**    **NO FRAMES**     **All Classes** |  |
| SUMMARY: NESTED \| FIELD \| CONSTR \| METHOD | DETAIL: FIELD \| CONSTR \| METHOD |  |


---


## sbrt.kernel.util Interface MutableProgressMonitor

**All Superinterfaces:**: ProgressMonitor

**All Known Implementing Classes:**: AbstractProgressMonitor, DiscreteProgressMonitor, PercentProgressMonitor

---

``` public interface MutableProgressMonitor extends ProgressMonitor ```

This interface is used to represent mutable progress montiors.

**Author:**
:   This interface was written and documented by
    Jeremiah Wright while in the Wagner lab.

---

| **Method Summary** | |
| --- | --- |
| `ProgressMonitor` | `asUnmodifiable()`             Returns this progress monitor as an immutable object. |
| `void` | `increment()`             Causes this progress monitor to increment an internal counter. |
| `void` | `resetCount()`             Causes this progress monitor to reset its internal counter to 0. |
| `void` | `start()`             Causes this progress monitor to start monitoring a duration of time. |
| `void` | `stop()`             Causes this progress monitor to stop monitoring a duration of time. |

**Methods inherited from interface sbrt.kernel.util.ProgressMonitor**

`getDuration, getProgress, started, stopped`

**Method Detail**

### increment

```
void increment()
```

:   Causes this progress monitor to increment an internal counter.

---


### start

```
void start()
```

:   Causes this progress monitor to start monitoring a duration of time.

    :   **Throws:**: `java.lang.IllegalStateException` - if `start()` was already called without a subsequent call to `stop()`.

---


### stop

```
void stop()
```

:   Causes this progress monitor to stop monitoring a duration of time.

    :   **Throws:**: `java.lang.IllegalStateException` - if `start()` was never called, or if `stop()` has already been called since the last call to `start()`.

---


### resetCount

```
void resetCount()
```

:   Causes this progress monitor to reset its internal counter to 0.

---


### asUnmodifiable

```
ProgressMonitor asUnmodifiable()
```

:   Returns this progress monitor as an immutable object. This allows
    the contents of this progress monitor to be queried without the
    possibility of modification.

    :   **Returns:**: this progress monitor as an immutable object.


---


|  |  |  |
| --- | --- | --- |
| **Overview** \| **Package** \| **Class** \| **Use** \| **Tree** \| **Deprecated** \| **Index** \| **Help** | |  |
| **PREV CLASS**   **NEXT CLASS** | **FRAMES**    **NO FRAMES**     **All Classes** |  |
| SUMMARY: NESTED \| FIELD \| CONSTR \| METHOD | DETAIL: FIELD \| CONSTR \| METHOD |  |


---
